# Elastin Structure, Synthesis, Regulatory Mechanism and Relationship With Cardiovascular Diseases

**DOI:** 10.3389/fcell.2021.596702

**Published:** 2021-11-30

**Authors:** Keke Wang, Xiangguang Meng, Zhikun Guo

**Affiliations:** ^1^Laboratory of Cardiovascular Disease and Drug Research, Zhengzhou No. 7 People’s Hospital, Zhengzhou, China; ^2^Henan Key Laboratory of Medical Tissue Regeneration, Xinxiang Medical University, Xinxiang, China

**Keywords:** elastin, matrix metalloproteinase, myocardial ischemia-reperfusion, atherosclerosis, atrial fibrillation

## Abstract

As the primary component of elastic fibers, elastin plays an important role in maintaining the elasticity and tensile ability of cardiovascular, pulmonary and many other tissues and organs. Studies have shown that elastin expression is regulated by a variety of molecules that have positive and negative regulatory effects. However, the specific mechanism is unclear. Moreover, elastin is reportedly involved in the development and progression of many cardiovascular diseases through changes in its expression and structural modifications once deposited in the extracellular matrix. This review article summarizes the role of elastin in myocardial ischemia-reperfusion, atherosclerosis, and atrial fibrillation, with emphasis on the potential molecular regulatory mechanisms.

## Introduction

Elastin is an extracellular matrix (ECM) protein responsible for the extensibility and elastic recoil of many vertebrate tissues, such as large arteries, heart valves, pulmonary tissues, skin, and certain ligaments and cartilages (Reichheld et al., 2019). Elastic fibers, which are the products of enzymatically cross-linked tropoelastin monomers and microfibrillar proteins, are composed of an insoluble polymerized elastin (*ELN*) core and microfibers located at the periphery (Yeo et al., 2011; Shin and Yanagisawa, 2019). Elastic fiber networks play an essential role in maintaining normal physiological functions, conferring elasticity, and recoiling to tissues and organs. In fact, both genetic and acquired cardiovascular diseases are associated with the insufficiency or disorganization of elastin and the breakage of elastic fibers (Cocciolone et al., 2018).

To better understand the relationship between elastin and the pathogenesis of cardiovascular diseases, we explored the structure, biosynthesis, and molecular regulation mechanism of elastin. Furthermore, we aim to provide new ideas for the treatment of these diseases.

## Structure and Distribution of Elastin

**FIGURE 1:**
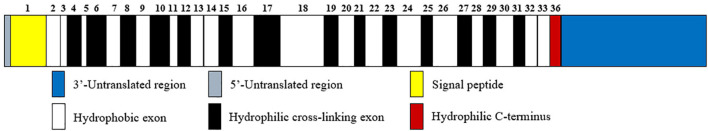
Human tropoelastin domain structure. Tropoelastin contains alternating hydrophobic (white rectangle) and hydrophilic cross-linking domains (black rectangle), and the width of the rectangles corresponds to the length of the domains. The number above the domains represents the sequence of exons that encode them. Among exons, exon 36 encodes a hydrophilic C-terminus (red rectangle) in addition to a 3′-untranslated region (blue rectangle).

Elastin is a resilient connective tissue protein found in the ECM of most vertebrate tissues, and it is an important part in the interstitium of tissues that undergo repeated physical deformations in the human body (Swee et al., 1995). Owing to its extensive crosslinked structures, elastin is a long-lived protein that degrades slowly in healthy tissues, with a half-life of about 70 years (Shapiro et al., 1991). As a polymeric molecule, elastin has hydrophobic and insoluble properties and can resist acid and alkali. In the presence of water, elastin exists in the form of a rubbery extension with a low elastic modulus (Partridge, 1969). In addition, over 75% of the sequence of elastin consists of just four non-polar amino acids, namely, glycine, valine, alanine, and proline (Vrhovski and Weiss, 1998). Like hydroxyproline-rich collagen, elastin contains about one-third glycine and approximately one-ninth proline. Tropoelastin, the soluble precursor of elastin with a molecular weight of about 60 kDa, is only encoded by a single gene, *ELN*, which is located on chromosome 7q11.1-21.1 with a size of 45 kb (Partridge, 1969; Swee et al., 1995; Tarakanova et al., 2018). The human *ELN* gene has 34 exons while the bovine gene contains 36 exons. Among them, exon 36 is highly conserved and codes for a hydrophilic C-terminus as well as a large 3′-untranslated region, in addition, the human gene does not have bovine exons 34 and 35 (Figure 1; Vrhovski and Weiss, 1998). Recently, alternate splicing variants of human tropoelastin mRNA transcripts have been reported. However, the specific generation mechanism of tropoelastin mRNA isoforms is still unclear (Reichheld et al., 2019).

Two major domains are found in tropoelastin: alternating hydrophobic regions and hydrophilic cross-link domains (Figure 1). The hydrophobic domains of tropoelastin are rich in non-polar amino acids, particularly glycine, valine, proline, and alanine, which often appear in repeats of 3–6 peptides, such as GVGVP, GGVP, and GVAP. The composition of this amino acid forms a hydrophobic interaction, which is required for the elastic property of the fiber. The hydrophilic cross-link domains are rich in alanine and lysine and are required for the lysyl oxidase-mediated formation of desmosine cross-links and are necessary to form insoluble elastin (Boyd et al., 1991; Vrhovski and Weiss, 1998).

Tropoelastin is known to be the most elastic and expansive monomer protein that can extend to eight times its length. Furthermore, the tropoelastin molecular shape is asymmetric. It extends into a coil-like structure in the N-terminal region of the molecule. Between molecules are supported by hinges, which act as a bridge to connect the N-terminal region to the C-terminal region of intercellular interaction, and then cross-linked by lysine oxidase (LOX) to form mature fibers (Baldock et al., 2011; Tarakanova et al., 2018).

Elastin is abundant in elastic tissues, and tissues rich in elastin include the aorta and major blood vessels (28–32% dry mass), the lungs (3–7%), elastic ligaments (50%), tendons (4%), and the skin (2–3%) (Uitto, 1979). Elastin and elastic fibers are also found within liver and myocardial tissues. Structurally, elastic fibers are primarily composed of extensively crosslinked elastin (>90%) and microfibrils rich in acidic glycoproteins and are organized into 8–16 nm bead-like fibrils of beaded appearance (Vrhovski and Weiss, 1998).

The elastic fibers in a healthy heart are mainly distributed in endocardium, visceral pericardium (epicardium), arterial wall, and cardiomyocytes and interweave with collagen fibers supporting elastic properties and strength. However, elastic fibers seldom form large fiber bundles like collagen fibers (Cunningham et al., 2006; Tsamis et al., 2013; Rodriguez and Tan, 2017). Studies showed that the length of mature elastic fibers was present at birth and maintain until adulthood in human heart parenchyma. The elastic fibers formed an irregular network in the myocardial interstitium in adults and a similar phenomenon was also observed in infants and children with thinner elastic fibers, however, in the elderly hearts the elastic fibers were thicker. What’s more, they also found the average length of elastic fiber was increased beyond the third decade of life (de Carvalho Filho et al., 1996).

## Elastin Biosynthesis and Assembly

**FIGURE 2:**
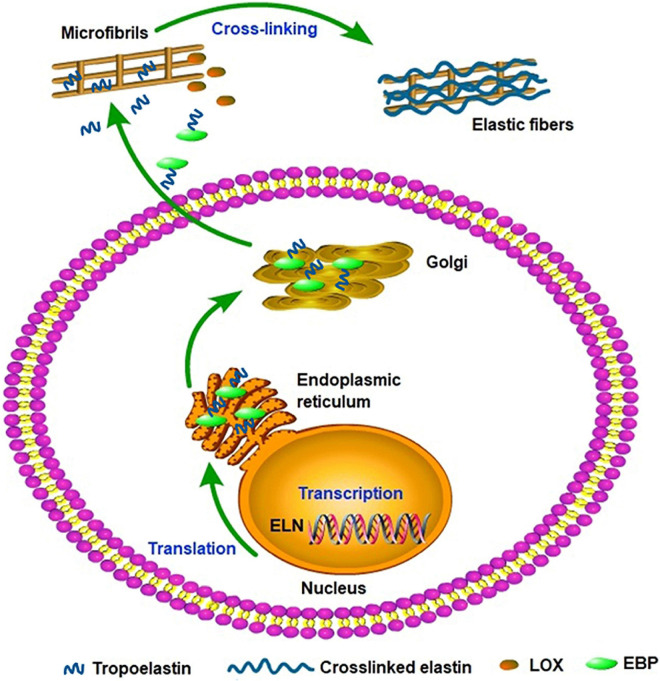
Tropoelastin secretory/assembly pathway. The synthesis of tropoelastin begins with the transcription of the ELN gene in the nucleus. In the endoplasmic reticulum, tropoelastin interacts with EBP and folds in structure. After the storage and transport of Golgi, the complex of EBP and tropoelastin is secreted to the cell surface for self-assembly. Then, tropoelastin dissociates from EBP and deposits onto the microfibril scaffold. In the presence of LOX, tropoelastin cross-links and eventually forms mature elastic fibers.

Elastin gene expression and protein synthesis occur within a narrow time frame from late embryonic development to the end of adolescence, producing no *de novo* elastin throughout the adult life of animals. Considering the elasticity and the approximately 70-year half-life of elastin, the limited time of expression and synthesis is enough for the protein to last a lifetime in most species (Cocciolone et al., 2018). In a stable state, the basal levels of elastin expression and breakdown remain low in adult tissues and cells isolated from adult tissues. *In vivo*, elastogenic cells including smooth muscle cells, fibroblasts, and endothelial cells can produce and secrete tropoelastin (Wise et al., 2009), moreover, we found that the co-expression of cardiac troponin T and elastin were detected in rat cardiomyocytes and rat myocardial tissues. Thus we speculate that the rat cardiomyocytes can secrete elastin and it may be involved in the potential energy of myocardial cells and the construction of intercellular elastic fibers (Han et al., 2019). In addition, elastogenesis begins with the transcription of *ELN*; subsequently, the mature mRNA of tropoelastin moves from the nucleus to the cytoplasm and then translated into tropoelastin in the rough endoplasmic reticulum (ER) (Swee et al., 1995). In the ER, tropoelastin interacts with elastin-binding protein (EBP) and protects tropoelastin from intracellular degradation and coacervation. Subsequently, the complex of EBP and tropoelastin is secreted to the cell surface to self-assembly. Tropoelastin monomers form multimers through the aggregation of their hydrophobic domains, and then deposit onto the microfibril scaffold. Under the modification of LOX, tropoelastin undergo extensive cross-linking and eventually form mature elastic fibers (Figure 2; Ozsvar et al., 2021). Recent studies suggest tropoelastin may follow an unconventional secretory pathway before transport to the cell surface. The assembly of elastin is thought to occur within the cell or at unique assembly sites on the plasma membrane, and after the self-aggregation, tropoelastin and other necessary assembly proteins are transported from the Golgi into an endosomal-like compartment along with LOX for crosslinking (Rabkin, 2017; Schrader et al., 2018; Kozel and Mecham, 2019; Shin and Yanagisawa, 2019). This may be a new perspective for better understanding the assembly mechanism of elastin. In the ER, tropoelastin also binds to several molecular chaperones, such as BIP and FKBP65 to assist with protein folding (Davis et al., 1998). The role of BIP is primarily to bind synthesized proteins, whereas FKBP65, an ER-resident peptidyl-prolyl *cis-trans* isomerase, modifies the kinetics of tropoelastin self-assembly in attenuating premature intracellular self-aggregation. The inherent ability of elastin to self-aggregate was described as a coacervation phenomenon and coacervation might play a critical role in the elastogenesis process which once impaired may induce elastin haploinsufficiency disorders including supravalvular aortic stenosis (Yeo et al., 2011). Studies showed that coacervation is an endothermic, entropically-driven process in which hydrophobic proteins separate from the solution as a second phase when the temperature is increased, and perhaps assisting in the alignment of lysine residues for crosslinking (Vrhovski et al., 1997). It has been reported that coacervation might order and align tropoelastin for crosslinking into the polymeric elastin matrix. And even if the lysine residues were modified the elastin polypeptides still were soluble without coacervation. Therefore, coacervation is important for the alignment of lysine residues present in the crosslinked region and the subsequent cross-link formation (Debelle and Tamburro, 1999; Keeley et al., 2002; Bellingham et al., 2003; Ostuni et al., 2007).

However, the precise molecular assembly pathway of the mechanical properties of elastin remains poorly understood.

## Molecular Regulatory Mechanism of Elastin

The dynamic regulation of elastin expression and formation in the development of various diseases involves a variety of molecules through transcriptional and post-transcriptional mechanisms, and it has engaged the interest of many researchers.

Matrix metalloproteinases (MMPs) are zinc-dependent endopeptidases that degrade ECM components, including elastin. MMPs can be activated by their proteolysis or other MMPs and the cleavage in amino-terminal prepeptide domains of serine proteases. Among MMPs, MMP-2 (gelatinase A), MMP-7 (matrilysin), MMP-9 (gelatinase B), and MMP-12 (macrophage elastase) have been shown to degrade elastin and its precursor tropoelastin. Moreover, recent studies reported that MMP-14 (MT1-MMP), has a similar cleavage effect on elastin, and MMP-14 also mediates MMP-2 activation (Rabkin, 2017; Miekus et al., 2019). By contrast, tissue inhibitors of MMP (TIMP), as endogenous inhibitors, prevent excessive ECM degradation caused by MMPs. Several studies have shown that the expression of MMP-2, MMP-7, MMP-9, MMP-12, and MMP-14 is significantly up-regulated in many cardiovascular diseases. However, changes in TIMPs levels are not entirely consistent (Johnson et al., 2014; Arvidsson et al., 2019; Deng et al., 2019; Kanagala et al., 2020; Merico et al., 2021; Morishita et al., 2021).

Cytokine-controlled elastin regulatory axes are involved in elastin expression regulation. As upstream regulators of MMPs and TIMPs, cytokines may cause an imbalance of the MMP/TIMP ratio resulting in altered myocardial (Kwak, 2013). Tumor necrosis factor-alpha (TNF-α), which is a pro-inflammatory cytokine, inhibits tropoelastin mRNA levels in skin fibroblasts, aortic smooth muscle cells, and lung fibroblasts. A previous study showed that TNF-α may induce vascular smooth muscle cell activation to produce MMP-2 and MMP-9 as well as inhibits TIMP activity and leads to elastin degradation *in vitro* (Kothapalli and Ramamurthi, 2010). TNF-α induced elastin degradation is associated with vasculitis caused by Kawasaki disease and vascular aneurysms, and the TNF-α deficiency prevents elastin degradation and aneurysm formation (Hui-Yuen et al., 2006). Like TNF-α, interleukin-1β (IL-1β) is a pro-inflammatory cytokine as well. In neonatal rat lung fibroblasts, IL-1β enhanced the release of elastases and down-regulated elastin transcription (Kuang et al., 2002). In the adult murine lungs, the production of IL-1β increased MMP-9 and MMP-12 expression and caused the disruption of elastin fibers in alveolar septa and airway fibrosis (Lappalainen et al., 2005). Conversely, transforming growth factor β (TGFβ), a potent profibrotic cytokine multiple, may have positive effects on tropoelastin transcription and stabilization and enhance tropoelastin mRNA expression. TGFβ1 is a member of the TGFβ superfamily, and several studies demonstrated that TGFβ1 inhibits MMP-2 and MMP-9 activation and stimulates TIMP-1 expression, thereby suppressing elastin degradation (Alvira et al., 2011; Sproul and Argraves, 2013). In addition, the overexpression of TGFβ1 stabilizes abdominal aortic aneurysms injured by inflammation and proteolysis to restore their ability to withstand arterial pressure (Dai et al., 2005). Heparin-binding epidermal growth factor-like growth factor (HB-EGF) is an EGF receptor ligand that downregulated elastin mRNA *via* activation of epidermal growth factor receptor and reduces matrix accumulation of elastin (Liu et al., 2003; Kwak, 2013). Furthermore, on the aging process of human mammary epithelial cells, a co-localization of MMP-7 and HB-EGF was demonstrated, and tropoelastin expression increased is associated with impaired HB-EGF and MMP-7 down-modulation. These observations suggest that MMP-7 and HB-EGF are interrelated in elastin regulation, but the specific mechanism remains unclear (Bertram and Hass, 2009).

**FIGURE 3:**
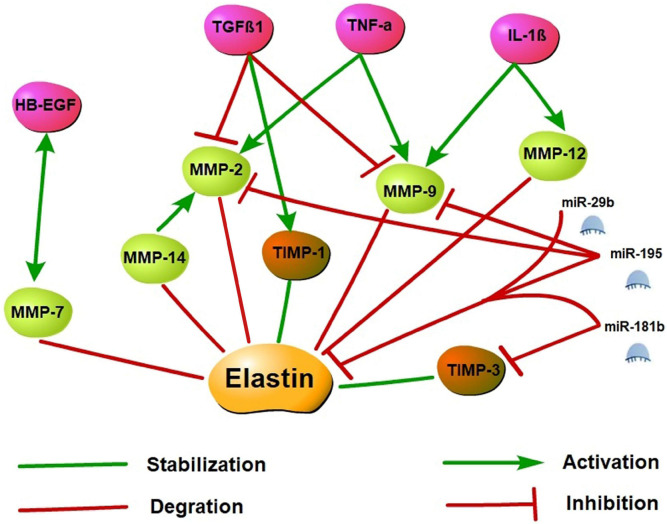
Elastin synthesis molecular regulatory networks. Elastin synthesis is dynamically regulated by a variety of molecules. Activated MMP may promote the degradation of elastin and its precursor tropoelastin, while the increase of TIMP may stabilize the synthesis of elastin. TNF-α, IL-1β, and HB-EGF may stimulate MMP activation and inhibit TIMP to induce elastin degradation. However, TGFβ1 may inhibit MMP activation and stimulate TIMP expression to suppress elastin degradation. Additionally, miR-29b, miR-195, and miR-181b are epigenetic regulatory molecules of elastin. These miRNAs may inhibit elastin expression by targeting the 3′ UTR of *ELN* mRNA, and restrained MMP and TIMP activity to involve in the regulation of elastin synthesis.

MicroRNAs (miRNAs) are small non-coding RNAs primarily involved in post-transcriptional regulation of gene expression by predominantly targeting the 3′UTR of mRNAs through destabilizing the transcript of mRNA or interfering in the translation process. Previous research has found that elastin expression is mainly regulated by the miR-29 family members (particularly miR-29b) and the miR-15 family members (particularly miR-195). Studies have demonstrated that miR-195 and miR-29b overexpression leads to a decrease in elastin. Using vectors carrying putative binding sites of miR-195 and miR-29b transfected into smooth muscle cells, previous works have shown that elastin is a direct target for both miRNAs. In *in vivo* experiments, miR-195 inhibited elastin and collagen expression and also restrained MMP-2 and MMP-9 activity, a degrading enzyme of ECM (Zampetaki et al., 2014; Kowara et al., 2017). In the heart, miR-29b is a key regulator of aortic dilatation, and its dysbiosis is also consistent with ECM remodeling after acute myocardial infarction (MI) (Kowara et al., 2017). Moreover, miR-29a inhibition can remarkably increase the *ELN* expression level in human cells. Therefore, the antagonistic effect of miR-29 may upregulate the *ELN* level in the case of elastically enhanced elastin dissolution or deficiency (Zhang et al., 2012). miR-181b is another epigenetic regulator of elastin gene expression. Using the mouse atherosclerosis models of ApoE^–/–^ and LDLR^–/–^, a study found that miR-181b inhibition substantially increased elastin and collagen expression levels through negative regulation of downstream target TIMP-3 expression and promoted a fibrotic response and consequent stabilization of existing plaques or aneurysms (Di Gregoli et al., 2017). Specific molecular regulatory mechanisms of elastin synthesis are shown in Figure 3.

Among other exogenous factors regulating elastin synthesis, vitamin C inhibits elastin gene expression and tropoelastin synthesis by changing mRNA stability and post-translational mechanism. As an ATP-dependent K^+^ channel opener, minoxidil can promote the expression of elastin mRNA in skin fibroblasts and smooth muscle cells. The regulatory mechanism of minoxidil on these cells is completed through Ca^2+^-ERK-dependent pathway *in vitro* (Daamen and Quaglino, 2019). Studies have shown that the blood pressure and functional arterial stiffness of *ELN*^±^ mice reduced to wild-type (WT) levels and the carotid and cerebral blood flows were restored when treated with minoxidil. Moreover, the expression of *ELN* was increased and 126 other matrix-associated genes were altered after the minoxidil treatment in *ELN*^±^ mice (Knutsen et al., 2018). Another research in aged mice found that the mRNA levels of tropoelastin, fibulin-5, and lysyl-oxidase were increased, and the expression of elastin was reinduced accompanied by the formation of newly synthesized elastic fibers. At the same time, the levels of advanced glycation end products were reduced with the chronic treatment of minoxidil (Coquand-Gandit et al., 2017). These results suggest that minoxidil may be involved in arterial remodeling and the improvement of cardiovascular disease by regulating the expression of elastin. And the treatment of minoxidil could be a potential strategy to improve arterial aging.

## Role of Elastin in Atherosclerosis

Atherosclerosis is a disease leading to the formation of plaques consisting of lipids, calcified regions, and immune and foam cells, along with vascular smooth muscles and endothelial cells in large- and medium-sized arteries (Fhayli et al., 2019). The altered homeostasis between synthesis and proteolysis of ECM proteins contributes to the development of atherosclerosis, and the alteration of elastin amounts, incorrect assembly, elastic fiber modification, and elastin fragments are associated with atherosclerosis. *In vivo*, damaged or degenerated elastic fibers are usually not repaired but are replaced by collagen and proteoglycan, making the arterial wall stiffer (Wahart et al., 2019). Moreover, a decrease in the ratio of elastin and collagen leads to arterial stiffness and arterial dilatation, further negatively affecting the normal function of blood vessels.

Calcification is a hallmark of late atherosclerosis (Speer and Giachelli, 2004). In the early stages, calcification is diffuse or punctate, but as the process progresses, calcium deposition, aggregation, and consolidation eventually form large flake calcium deposits. In addition, the diffuse calcification of vascular intima is also related to some components of ECM, especially matrix vesicles. Matrix vesicles come from osteoblast, cartilage cell, odontoblast and budded from cell, then apart from the cell to form self-governed organelle. Matrix vesicles provide suitable nucleation and micro-environment to calcium crystal, and the elastin in the vascular wall provides a network scaffold for calcium crystal deposition. Moreover, the elastic fibers in the ECM may take part to the arterial wall calcification. Electron microscopy and histochemistry revealed two different types of elastic fiber calcification in human atherosclerotic plaques. No structural changes in elastin are detected in type I calcification, whereas structural changes in elastin occur earlier in type II calcification (Bobryshev, 2005). Khavandgar et al. (2014) demonstrated that in matrix Gla protein (MGP)-deficient and *ELN* haploinsufficient (*MGP*^–/–^; *ELN*^±^) mice, the severity of medial calcification is elastin dependent, and elastin haploinsufficiency has significantly reduced arterial mineral deposition compared with *MGP*^–/–^ mice. This experiment suggests that the content of elastin might be an important determinant of medial calcification. Degradation of elastin in the arterial wall is also a typical feature of atherosclerosis. Elastin-derived peptides (EDPs), the degradation products of elastin, display a wide range of biological activities, and EDPs have previously been shown to be related with atherosclerosis. Studies have shown that serum concentrations of EDP (S-EDP) are higher in atherosclerotic patients compared to healthy subjects, and concentrations of elastin-derived peptides were statistically significant higher in children from families at high risk for atherosclerosis (Gmiński et al., 1991). However, Petersen et al. (2002) suggested that increased concentrations of S-EDP were associated with aneurysmal and ulcerative, but not occlusive, manifestations of atherosclerosis. The presence of EDPs in serum may reflect the intensity of the elastic dissolution process in atherosclerotic lesions. Inflammatory cytokines, growth factors, oxidative stress, and hypoxia produced in the process of atherosclerosis lead to the breakdown of elastin and the production of elastokines (Bobryshev, 2005; Wahart et al., 2019). EDPs can dissolve in the blood and spread out in the whole body from disrupted plaques, which may cause harmful complications such as plaque instability. Therefore, we speculate that increased concentrations of EDPs may be a potential risk factor for atherosclerotic complications. From the results, we speculate that the structural alterations of elastin and elastic fibers occurring in the early stages of atherosclerosis can induce the calcified deposits formed on the base of pre-existing structures with the deposition of unesterified cholesterol. Besides that, EDPs, the degradation products of elastin actively participate in the pathogenesis of atherosclerosis such as low-density lipoprotein oxidation and vascular calcification and accelerate the progression of the disease.

In addition, elastin is also affected by glycosylation and carbamylation (Gorisse et al., 2016) which may exacerbate the progression of chronic kidney diseases by modulating the hardness of elastin. However, whether these modifications have a direct effect on atherosclerosis progression remains to be demonstrated.

As we summarized above elastin can be cleaved by elastinases and MMPs, and the excessive production of elastin fragmentation may have a deleterious effect on atherosclerosis progression. Thus, chemical compounds designed to bind to elastin and simultaneously inhibit elastolytic activities might be new therapeutical strategies. Selective murine MMP-12 inhibitor has been reported by the group of Dive, MMP-12 is associated with plaque progression and instability. The MMP-12 inhibitor significantly reduced atherosclerotic plaque area and delay atherosclerosis development in ApoE^–/–^ mice (Quillard et al., 2011).

## Role of Elastin in Myocardial Ischemia-Reperfusion Injury

Atherosclerosis can cause progressive stenosis of the coronary artery, which makes the blood and oxygen supply of the heart insufficient, resulting in ischemia of downstream tissues. Generally, ischemia-reperfusion can only restore the blood flow of ischemic tissue, cannot restore tissue, organ function, on the contrary, aggravate tissue, organ dysfunction, and structural damage called ischemia-reperfusion injury. The mechanisms involved in myocardial ischemia-reperfusion injury may include reperfusion triggered oxidative burst, calcium overload, and mitochondrial damage, which together induce cardiomyocyte apoptosis and necrosis, leading to irreversible damage (Yellon and Hausenloy, 2007).

When ischemia-reperfusion occurs, myocardial interstitial components are reconstructed while cardiomyocytes are damaged. However, such reconstruction is rarely reported in recent literature. Myocardial tissues on normal state of physiology contain a small number of elastic fibers, which are primarily derived from fibroblasts and smooth muscle cells. Research in rats shows that fibrosis occurs in the infarcted area after suffering myocardial ischemia (MI), forming cicatricial tissues, which decrease the elasticity of the ventricular wall and influence heart function. And the expression of recombinant elastin contributes to maintaining the elasticity of MI tissues (Mizuno et al., 2005). Our studies have proved that the increase in collagen fiber content and the decrease in elastic fiber content are the pathological basis of the enhanced tissue hardness and the decrease of elasticity in myocardial scar tissues located in the infarcted area in Sprague–Dawley rats (Yu et al., 2018). Therefore, elastin is believed to play an important role in preventing regional expansion of cicatricial tissues in the infarcted area, left ventricular enlargement, and improvement of ejection fraction (Mizuno et al., 2005).

Researchers examined the images of elastin and mature elastin fibers using an elastin/tropoelastin-specific contrast agent (i.e., Gd-ESMA) by constructing myocardial ischemia model in mice, and found the presence of tropoelastin and mature elastin in the infarct scar regions, and this presence was associated with improved ejection fraction of heart after MI *in vivo* (Protti et al., 2015). Mizuno demonstrated that increasing the expression of recombinant elastin within the myocardial scar by transfecting elastin gene into rat endothelial cells can reduce scar expansion and preserve heart function after MI (Mizuno et al., 2005). Lichtenauer et al. (2011) showed that in the rat myocardial infarction model, the scar tissue gradually formed after myocardial infarction, in which the expression of elastin protein increased, but this myocardial remodeling can be blocked by the intravenous or intramyocardial injection of apoptotic leukocyte suspension. Compared with collagen in fibrotic tissues, a high proportion of elastin can change the composition of myocardial scar to maintain the elasticity of the infarcted heart and improve cardiac function, thereby avoiding progressive heart failure. Therefore, the increased synthesis and composition of elastin fibers is a decisive mechanism for maintaining infarct scar structure and elasticity to avoid the gradual loss of cardiac biomechanical functions. However, none of these studies has involved the specific molecular regulatory mechanism of elastin in the disease process, which needs further study.

The fragmentation of elastin by elastinolytic enzymes named EDP is a hallmark of aging tissues. This aging marker was also observed in myocardial tissue during ischemia. EDPs were considered to have detrimental effects on the cardiovascular system, showing chemotactic activity against neutrophils, inducing T-Helper-type1 polarization in human blood lymphocytes, secretion of cytokines and enzymes in fibroblasts and aortic smooth muscle cells (Debret et al., 2005). Robinet et al. (2007) have shown that EDPs interaction with S-Gal (elastin binding protein) can protect the heart against ischemia/reperfusion injury by triggering nitric oxide (NO) release and activating PI3-kinase/Akt and ERK1/2 in human coronary endothelial cells (HCAECs) and rat neonatal cardiomyocytes (RCs). But researchers later found that such protective effect might be lost with aging in rats. The elastin receptor complex (ERC) is a heterotrimer that is composed of elastin binding protein, protective protein or cathepsin A (PPCA), and membrane-bound neuraminidase 1 (Neu-1) and derived from the lysosomal β-galactosidase(β-Gal) complex. S-Gal is a spliced variant of β-Gal and this splicing form, which retains the ability to bind galactose or lactose and lacks enzyme activity, can bind to EDPs as antagonists (Maurice et al., 2013).

At present, blocking the combination of elastic factor and ERC may be a new treatment strategy. For example, a peptide that mimics the S-Gal sequence is designed to limit the harmful effects of elastin fragmentation at the molecular level.

Beyond that, both NO and reactive oxygen species (ROS) are involved in the myocardial ischemia-reperfusion injury or neurovascular protection after ischemic stroke, depending on their concentrations. Elastin-Derived Peptide VGVAPG decreased the expression of eNos, iNos, and nNos mRNA and proteins in mouse astrocytes *in vitro*. The VGVAPG peptide also reduces the production of NO in cells and increases the production of ROS. Therefore, it is speculated that VGVAPG peptide is involved in activating the survival/healing pathway of astrocytes (Szychowski and Gmiński, 2019).

Reactive oxygen species are generally considered as toxic by-products of aerobic metabolism and the main cause of macromolecular damage, and reperfusion is associated with the outbreak of ROS production. Previous studies have shown that ischemic oxidative stress is a precursor of cell death in the process of myocardial cell reperfusion (Robin et al., 2007). Therefore, whether EDPs generation plays a beneficial protective role with myocardial ischemia-reperfusion injury is still an unsolved problem.

## Role of Elastin in Atrial Fibrillation

Atrial fibrillation (AF) is a common clinical arrhythmia that affects atrial cardiomyocytes and interstitials. One of the generally recognized factors that affect the development and maintenance of AF is cardiac remodeling. Experimental models showed that AF leads to a variable degree of structural remodeling of atrial tissues that in turn promotes further maintenance of arrhythmias (Wakili et al., 2011). However, limited clinical evidence is available to support this view.

Tissue interstitial fibrosis, the important morphological basis for AF, is also an important marker for structural reconstruction. According to morphological observation, myocardial fibrosis is the increase of ECM proportion, especially collagen (protein) fiber deposition. As the important components of the atrial wall, collagen, elastin, and reticular fibers determine its mechanical properties, such as compliance (Fomovsky et al., 2010). Smorodinova et al. (2015) showed that the contents of interstitial elastin, collagen I, and collagen III in atrial tissues of patients in the AF and SR groups were similar, and no remarkable difference was observed in vascular endothelial growth factor (VEGF) expression and microvascular density. However, the expression of elastin, collagen I, collagen III, and VEGF in the right atrium tissue was remarkably higher than that in the left atrium tissue, and the microvascular density of the left atrium myocardium was higher than that of the right atrium. Therefore, interstitial fibrosis and other morphological changes in atrial tissues are associated with structural heart diseases but not with AF. However, regional differences in these institutions in the left and right atria warrant further study. Recent research showed that in animal models, atrial stretch leads to increased atrial fibrosis, thereby inducing regional remodeling and slow conduction. Atrial stretch causes ANP release, calcium overload, calcineurin activation, AT1 receptor involvement, and altered levels of MMP and TIMP. Moreover, atrial stretch is associated with tissue turnover and remodeling. Furthermore, atrial distraction can induce inflammation and fibroblast proliferation, collagen elaboration, and scar formation, and this process is TGF-β1 mediated (Yang et al., 2017). However, the specific mechanism of elastin and AF is not clear, so it is necessary to explore further.

## Conclusion

Many studies on elastin structure and biosynthesis have been conducted, but its specific method of assembly has not been determined thus far. At present, studies on elastin expression regulation often tend to focus exclusively on cell experiments or animal models and thus cannot accurately simulate the complex internal environmental homeostasis of humans. This homeostasis is often accompanied by alterations of elastin expression levels and structures in many cardiovascular diseases. However, its accurate mechanism of action is complex and remains inconclusive. It’s necessary for us to better understand how enzymes associated with inflammation lead to structural remodeling and loss of functional integrity of elastic fibers in heart diseases. And it’s also important to seek biomarkers of elastic fiber injury to realize the diagnosis and treatment in the early stages of diseases. Therefore, the comprehensive action of elastin and its various regulatory molecules must be explored to establish strategies for the prevention and treatment of related diseases.

## Author Contributions

ZG conceived the study. XM prepared the figures. KW drafted the manuscript. All authors contributed to the article and approved the submitted version.

## Conflict of Interest

The authors declare that the research was conducted in the absence of any commercial or financial relationships that could be construed as a potential conflict of interest.

## Publisher’s Note

All claims expressed in this article are solely those of the authors and do not necessarily represent those of their affiliated organizations, or those of the publisher, the editors and the reviewers. Any product that may be evaluated in this article, or claim that may be made by its manufacturer, is not guaranteed or endorsed by the publisher.
